# Preparation of Eugenol-Containing Epoxy–Siloxane Polymers for Coating Materials

**DOI:** 10.3390/polym18192313

**Published:** 2026-09-22

**Authors:** Pavel V. Lukin, Alisa A. Shmakova, Nikita Yu. Budylin, Aleksey V. Shapagin, Yulia V. Bilichenko, Vyacheslav V. Kireev, Mikhail A. Soldatov

**Affiliations:** 1 Department of Chemical Technology of Polymer Composite Paints and Coatings, Mendeleev University of Chemical Technology, Miusskaya Sq. 9, 125047 Moscow, Russia; pavel.lukin123@mail.ru (P.V.L.); alisa.sh2004@yandex.ru (A.A.S.); 2 Laboratory of Structure and Morphology Research, Frumkin Institute of Physical Chemistry and Electrochemistry, Russian Academy of Sciences, 31-4, Leninsky Prospect, 119071 Moscow, Russia; budylin_nikita@mail.ru (N.Y.B.); shapagin@mail.ru (A.V.S.); 3 Department of Chemical Technology of Plastic Materials, Mendeleev University of Chemical Technology, Miusskaya Sq. 9, 125047 Moscow, Russia; bilichenko.iu.v@muctr.ru (Y.V.B.); kireev.v.v@muctr.ru (V.V.K.)

**Keywords:** eugenol, oligoorganosiloxanes, hydrosilylation, epoxy–siloxane polymers, film-forming materials, coatings

## Abstract

In this work, an eugenol-containing polysiloxane was synthesized by means of a hydrosilylation reaction between eugenol and polymethylhydridesiloxane. It was found from the laser interferometry that the obtained polymer can be compatible with some epoxy resins and that the compatibility increases with epoxy resin molecular weight. Thus, the synthesized polymers are fully compatible with epoxy resins with molecular weights of less than 700. The compositions of epoxy resin and the synthesized eugenol-containing siloxane polymer can be cured at temperatures higher than 180 °C and are able to form coatings with good physico-mechanical properties. It was found that the addition of a siloxane polymer leads to an increase in thermal stability, and that the T_d5%_ for all compositions was higher than 340 °C. In addition, the obtained coatings have contact angles of 83–92°, which is higher than that for epoxy resins cured with traditional curing agents.

## 1. Introduction

Eugenol (4-allyl-2-methoxyphenol) is a natural phenol contained in various plants like carnation, cinnamon, oregano, etc. [1]. It is of great interest to use eugenol for the preparation of various monomers and polymers due to the presence of three types of functional groups in its structure [2,3,4,5,6]. For example, the introduction of eugenol in a polymer structure can impart antibacterial properties and thermal stability [7,8]. As for organosilicon polymers, reactions such as thiol-ene addition [9,10], hydrosilylation [11,12,13,14], and Piers–Rubinsztajn [15,16] can be used for functionalization with eugenol as well as their combinations, which allows us to obtain cross-linked eugenol-siloxane polymers [17,18].

On the other hand, the introduction of siloxane into the chemical structure of epoxy polymers may lead to an increase in the hydrophobicity and thermal stability of the final cross-linked polymer. Thus, in a number of works, various organosilicon polymers were used for such modifications [19,20,21,22]. All the works, dedicated to eugenol-containing epoxy–siloxane polymers, might be divided on several types, depending on the structures of the used and synthesized compounds. Several works are dedicated to the addition of eugenol through a hydrosilylation reaction to tetramethyldisiloxane. Such compounds can then be used as phenolic curing agents for multifunctional epoxy resins like epoxy novolac resins [23], or they can react with epichlorohydrin to form siloxane-based epoxy resin [24,25,26]. Due to the presence of reactive protons in eugenol rings, the obtained compounds can react with formaldehyde to obtain novolac-like oligomers that are able to cure epoxy resins [27]. A similar approach was used when tetramethylcyclotetrasiloxane was used instead of tetramethyldisiloxane [23,28,29], which was due to higher functionality providing a higher cross-linking degree and in turn higher thermal stability and physico-mechanical properties. Several works are dedicated to the synthesis of eugenoxy-substituted silanes via the reaction of eugenol with chlorosilanes [30,31]. Then, the allyl groups of eugenol fragments can be epoxidized to form epoxy-functionalized silanes.

Despite these aforementioned works, dedicated to low-molecular-weight eugenol-containing organosilicon compounds, the number of works where polyfunctional eugenol-containing polysiloxanes were used is quite low. But such high functionality might provide enhanced physico-mechanical and other operating properties, as was mentioned for tetramethylcyclotetrasiloxane derivatives. In the work of Chen et al. [32], a eugenol-modified siloxane polymer was obtained by means of hydrosilylation of polymethylhydridesiloxane with the mixture of eugenol and vinyltriethoxysilane. Such a copolymer was further used in the curing of epoxy resin and improved the thermal stability of the final polymeric coatings.

Another problem for epoxy–siloxane polymer compositions might be the compatibility of epoxy and siloxane components. In our previous work, the compatibility of eugenol-containing oligoorganosilsesquioxane with epoxy resin was studied, and it was shown that the components are compatible at room temperature or higher and are able to form cross-linked polymers at temperatures above 140 °C [33].

Thus, it is of great interest to obtain novel compositions based on eugenolized oligo- or polysiloxanes and epoxy resins, as well as to study how their compatibility might influence the proeprties of the final materials. Thus, the linear eugenol-functionalized oligo- and polysiloxanes are promising compounds, which might be used as plastisizers and hydrophobic additives as well as curing agents for epoxy resins to obtain eco-friendly and multifunctional film-forming materials. In this work, we have synthesized in a simple way eugenol-containing epoxy–siloxane compositions, based on commercially available polymethylhydridesiloxane and epoxy resins.

## 2. Materials and Methods

### 2.1. Materials

Eugenol was purchased from VladMiVa company (Belgorod, Russia), polymethylhydridesiloxane (PMHS) was purchased from «XJY Silicones» (Jiujiang, China), and the Karstedth catalyst was purchased from «ABCR» (Karlsruhe, Germany). Four types of epoxydiane resins were purchased from Chimex Ltd. (St. Petersburg, Russia). The trademarks and main properties of the used epoxy resins are given in Table 1. Toluene was dried on phosphorus pentachloride and was freshly distilled before synthesis. All the other chemicals were used as received.

### 2.2. Synthesis of Eugenol-Containing Polysiloxane (ES)

The ES was synthesized by hydrosilylation of PMHS with eugenol (Scheme 1). Eugenol and toluene were charged in a three-necked round-bottom flask, equipped with a magnetic stirrer, a reflux condenser and a additional funnel. Then, the Karstedth catalyst at 0.4 wt. % of the weight of the reaction mixture was added, and then the solution of PMHS in toluene was added dropwise to prevent the temperature of the reaction mixture from exceeding 50 °C. Then, the reaction mixture was stirred for 3 h at room temperature. Then, toluene was removed on a vacuum rotary evaporator at 50 °C. The final polymer was purified by column chromatography on silica gel with the use of dichloromethane as an eluent.

### 2.3. Preparation of Eugenol-Containing Epoxy–Siloxane Compositions and the Coatings Based on Them

The compositions were obtained on the base of ES and ED-20 or E-41 by mixing the components at 125 °C for 3 h with the use of an overhead stirrer. Concentrations of the components are given in Table 2. The obtained compositions were dissolved in ethyl cellosolve to obtain 40 wt. % solutions. Then, 0.2 wt. % of the surface additive BYK-331 (BYK-Chemie GmbH, Wesel, Germany) was added for better formation of the final coatings.

The coatings were obtained by casting the obtained solutions onto metal or glass substrates with the use of a pneumatic spray gun or with the use of a frame applicator. Then, the coatings were cured at 180 °C for 3 h.

### 2.4. Characterization Methods

FTIR spectra were recordered on “SPECTRION FT” (Shenzhen ION Engineering Technologies Ltd., Shenzhen, China) spectrometer in a range from 4000 to 350 cm^−1^.

^1^H and ^13^C NMR spectra were obtained with the use of a “Bruker WB Avance III” (Karlsruhe, Germany) spectrometer at frequencies of 600.13 and 150.92 MHz, respectively, with the use of CDCl_3_ and (CD_3_)_2_CO as deuterated solvents.

Molecular-weight characteristics were determined by gel-permeation chromatography (GPC) with the use of a “Stayer” chromatograph (Akvilon Ltd., Podolsk, Russia) equipped with column Phenogel (1–500 kDa) at 60 °C and with an eluent rate of 1 mL/min. DMF was used as the eluent.

Microstructure and elemental analysis of surface were measured on a scanning electron microscope Thermo Fisher Scientific Quattro C (Brno, Czech Republic) at an accelerating voltage of 30 kV, a probe current of 0.98 nA in high vacuum, and with pre-spraying of a 10 nm thick layer of carbon. The carbon layer was sputtered on a compact sputtering machine, Safematic CCU-010 LV (Zizers, Switzerland). To determine the elemental composition of the powder particles, energy-dispersive X-ray microanalysis (EDX) was performed in high vacuum mode using an analytical X-ray detector, UltraDry, from Thermo Scientific (Brno, Czech Republic).

The study of solubility in binary systems was carried out by optical interferometry on an ODA-2 diffusion interferometer (IPCE RAS, Moscow, Russia). Epoxy was placed in a diffusion cell between glasses, the inner sides of which were coated with a translucent layer of the Ni-Cr alloy. An angle of ≤2° was set between the glasses, which was necessary for the appearance of an interference picture when passing a laser beam with λ = 635 nm. Then, the space between the glasses was filled with a copolymer. In order to obtain information about phase equilibria in systems, studies were carried out in the mode of stepwise heating and cooling with a step of 10 °C in the temperature range from 160 to 20 °C. At each stage, the system was thermostated to a state of equilibrium, and interferograms were recorded. The moment of contact of the components was considered the beginning of the interdiffusion process. The interference picture in the interdiffusion zone was characterized by the curvature of isoconcentration bands in accordance with the change in the refractive index in the mixture of the studied components. By processing the mutual diffusion zones of the interferograms, concentration profiles were obtained. A smooth change in concentration in the diffusion zone from one component to another indicated the homogeneity of the system at a given temperature. Concentration jumps in the profiles determined the compositions of coexisting phases corresponding to the points of the binodal curve of the phase diagram.

Thermogravimetric analysis (TGA) was performed on DZ-TGA101 analyzer (Nanjing Dazhan Testing Instrument Co., Ltd., Nanjing, China) in air atmosphere and at a temperature range from room temperature to 700 °C, with a heating rate of 10 °C/min.

The contact angle values were obtained by the sessile drop method using an FM 40 Mk 2 EasyDrop device (Krüss GmbH, Hamburg, Germany). The contact angles were measured after 30 sdripping on the surface of the coating.

Adhesion of the coating was measured by a cross-cut test according to ISO 2409:2020 [34] and by a pull-off test according to ISO 4624:2002 [35].

Impact strength of coating was determined by ISO 6272-1:2011 [36].

Coating bending strength was measured according to ISO 1519:2011 [37].

Coating hardness was measured according to ISO 1522-2006 [38], based on the damping of a König pendulum on the coating.

The anticorrosive properties of the coatings were evaluated by the static exposure of two coated steel plates to a 3% NaCl solution or distilled water for 120 h according to ISO 2812-1:2017 [39] and ISO 2812-2:2018 [40]. Salt-spray tests of the coatings were performed according to ISO 9227:2017 [41] with the use of a salt-spray chamber ASCOTT Premium S450ip (Ascott Analytical Equipment Limited, Tamworth, UK).

## 3. Results and Discussion

At the first stage, the eugenol-containing polysiloxane ES was synthesized by a hydrosilylation reaction between eugenol and PMHS. On the ^1^H NMR spectra (Figure 1), one can find that the signals of initial PMHS Si-H protons at δ ≈ 4.7 ppm as well as the signals of double bonds of eugenol at δ ≈ 5.2 and 6.1 ppm are almost abscent. Moreover, the new signals at δ ≈ 0.6, 1.7 and 2.5 ppm appear, corresponding to -Si-CH_2_-, -Si-CH_2_-CH_2_- and -Si-CH_2_-CH_2_-CH_2_- respectively, which indicates a successful reaction.

Analysis of the ^13^C-NMR spectra also shows that in ES, one can observe the appearance of new signals at 18 and 26 ppm, corresponding to aliphatic carbon atoms, and the complete disappearance of signals at 115 and 137 ppm, corresponding to the double bonds of the allyl group.

In FTIR spectrum of ES (Figure 2) one can see signals at 1000 and 1510 cm^−1^ corresponding to Si-O-Si and C-C bonds of aromatic ring respectively. On the other hand the peaks of Si-H and CH=CH bonds are absent.

According to the GPC analysis (Figure S2), the obtained ES has a wide and almost monomodal molecular weight distribution with low amount of low-molecular-weight fractions and has the following molecular-weight characteristics (relatively to polystyrene standards): M_n_ = 6600, M_w_ = 22,400, M_w_/M_n_ = 3.4.

On the next stage, the compatibility of the synthesized ES with epoxy resins was studied by laser interferometry. In the case of the resin ED-20, which has the lowest molecular weight, one can observe full compatibility in the temperature range from 22 to 160 °C (Figure 3).

Similar behavior is observed in the case of resin E-40, having a higher molecular weight (Figure 4). In the interferrograms of ED-20 and E-40 systems, one can see the absence of a phase boundary between the components and bends of interference lines, indicating the change in refractive indexes due to a mutual interdiffusion of components of the binary systems. This allows us to conclude that there is complete mutual compatibility between the components in the entire studied temperature range.

When the molecular weight of the epoxy resin increases, the compatibility with ES decreases until reaching full incompatibility (Figure 5). As one can observe, systems with high-molecular-weight resins ED-8 and E-41 have clear phase boundaries in the whole studied temperature range, which separates areas of ES dissolved in epoxy resin and epoxy resin dissolved in ES. The low compatibility of the components does not allow us to determine the systems as systems with lower or upper critical solution temperature.

For the further tests, we used ED-20 resin, which is completely compatible with ES and E-41 resins, which is contradictorarily incompatible with ES in studying how compatibility may affect on the properties of the final coating materials. It was supposed that during the mixing of the components at elevated temperatures, they might react in the same way as how phenolic resins react with epoxy resins. Such a deeper polyaddition reaction at high temperatures leads to the formation of cross-linked polymers (Scheme 2).

After mixing the ES and epoxy resin, the obtained compositions were studied by NMR spectroscopy (Figure S3). It was shown that the ration of integral intensities of protons in epoxy resin remains almost the same in all compositions as well as in the initial epoxy resin. These results show that no reaction between epoxy and phenol groups take place when the components were mixed at 125 °C. The GPC curves of the compositions also show only the peaks of ES and the epoxy resin (Figure S4).

The obtained compositions were cured at 180 °C to obtain coatings or unsupported films. On the FTIR spectra of the films of ED-20:ES-60 and ED-20:ES-70 (Figure 6), one can observe the disappearance of a peak at ~910 cm^−1^ related to asymmetric valent vibrations of the C-O-C bond in an epoxy ring, which confirms a successful cross-linking reaction. On the spectrum of ED-20:ES-50, in turn, one can observe a residual small peak at ~910 cm^−1^, which indicates that when the content of ES in the composition is 50 wt. % and less, the amount of phenol groups is not enough to react with all epoxy groups.

In the case of E-41, its incompatibility with ES and the phase separation of the components do not provide a uniform cross-linking reaction in the bulk of the system despite the high reactivity of phenol and epoxy groups. Thus, the components mostly react with each other locally on the phase boundary. The FTIR spectrum of E-41:ES-20 (Figure 7) demonstrates the presence of an epoxy group peak at 910 cm^−1^, indicating the unsuccessful or non-complete reaction of the components.

SEM analysis with elemental mapping of the cured films (Figure 8) shows, that in ED-20:ES compositions silicon atoms are uniformly distributed on the whole studied surfaces. As for the E-41:ES-20 composition, one can see a clear phase delamination where one phase contains silicon atoms while another one almost does not contain silicon.

The thermal stability of the obtained cured films was studied by TGA in air atmosphere (Figure 9, Table 3). Here, one can see that for all samples, the degradation temperatures T_d5%_ are in range of 340–350 °C, which is comparable with the other thermally stable epoxy resins, cured with aromatic amines, anhydrides, phenols, etc. More interesting are the char yield values. According to the results, char yield at 700 °C for E-41-ES-20 is much lower than that for the other systems, based on the ED-20 resin, which is explained by poorer cross-linking degree. The ED-20:ES-60 system gives the highest char yield value, which allows us to conclude that this is an optimal ratio of ED-20 and ES, giving the highest cross-linking degree.

The final coatings were obtained on steel or tin plates, depending on the further characterization method. Coatings, based on the ED-20 resin, are transparent and smooth, which indicates that the obtained composition possess good film-forming properties (Figure S5). Physico-mechanical and physico-chemical properties of the coatings are given in Table 4. The coatings exhibit water contact angles in the range of 83–92°, depending on the composition (Figure S6). These values are higher than for the epoxy coatings, cured with traditionally used curing agents like amines or anhydrides (which are usually in the range 60–75°) due to the presence of hydrophobic methylsilyl units in ES structure. Thus, the use of ES as a cross-linker allows us to obtain epoxy coatings with water-repellent properties.

According to results from Table 4, the coatings possess good physico-mechanical properties. High elasticity of the coatings is explained by the high flexibility of siloxane units and the optimal length of segments between cross-linking points. Among the ED-20 compositions, the hardness has maximum value for the composition ED-20:ES-60, which additionally proves that this ratio of the components provides the highest cross-linking degree.

Anticorrosive performance of the coatings is proved by low corrosion spreading in the salt-spray test (Figure S7). Also, the coatings are stable for a long time of immersing in distilled water and NaCl solution, which also proves a good anticorrosive performance.

Thus, based on the results, the obtained coatings possess high-performance properties and can provide good and complex protection of metal substrates.

## 4. Conclusions

In conclusion, the linear eugenol-containing siloxane polymer was synthesized and studied. For the first time, the compositions of the synthesized polymer and epoxy resins were obtained and studied. It was shown that the synthesized organosilicon polymer is compatible with low-molecular-weight epoxy resins and this compatibility decreases with the increase in epoxy resin molecular weight. The obtained eugenol-containing polysiloxane can be a promising curing agent to obtain high-performance coatings with good hydrophobicity and anticorrosive properties. This work opens perspectives in the preparation of functional epoxy–siloxane coatings. The main research issue consists of the compatibility of the composition components. Thus, future works are aimed at preparing eugenol-containing siloxane polymers and copolymers, which could be more compatible with high-molecular-weight epoxy resins. Such compatibility might be achieved by lowering the molecular weight of polysiloxane or by preparing siloxane copolymers having phenyl groups, thus providing good compatibility with high-molecular-weight epoxy resins.

## Figures and Tables

**Scheme 1:**
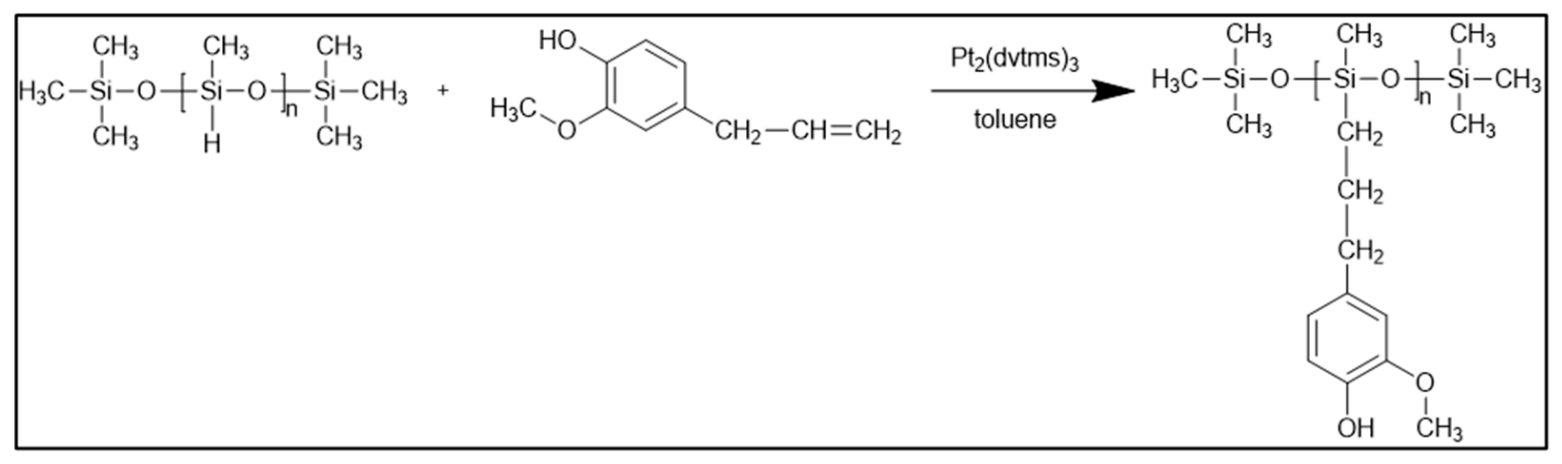
Hydrosilylation of PMHS with eugenol.

**Figure 1:**
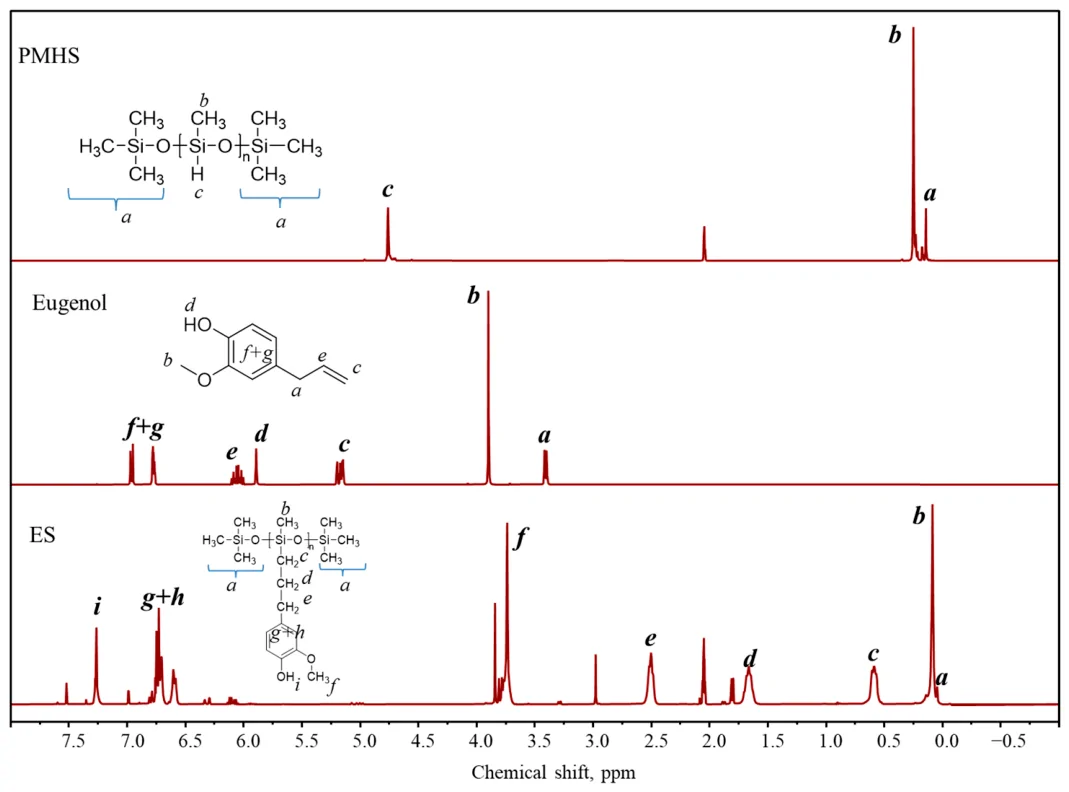
^1^H-NMR spectra of initial PMHS and eugenol and the final ES.

**Figure 2:**
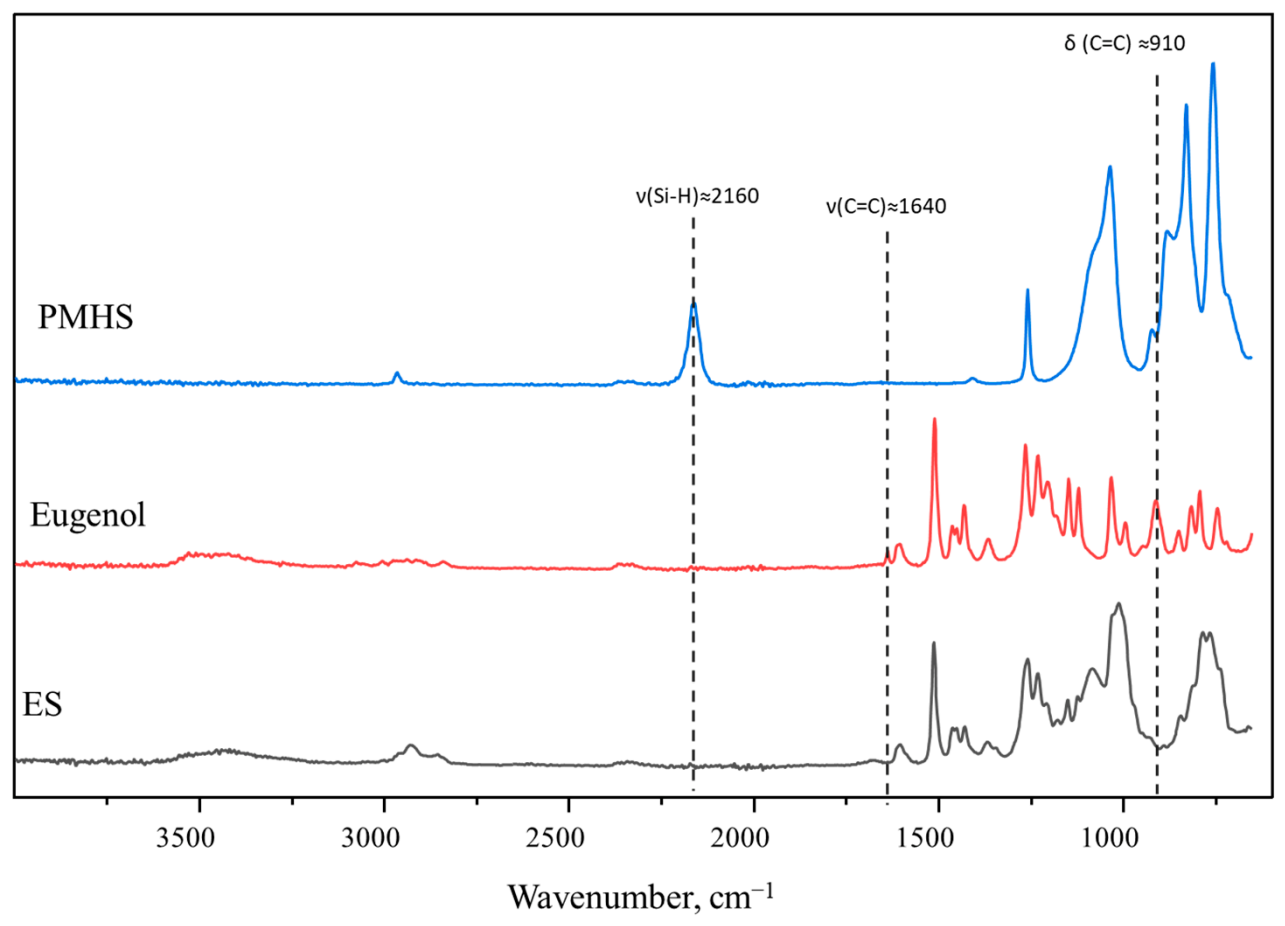
FTIR spectra of initial PMHS, eugenol and final ES polymer.

**Figure 3:**
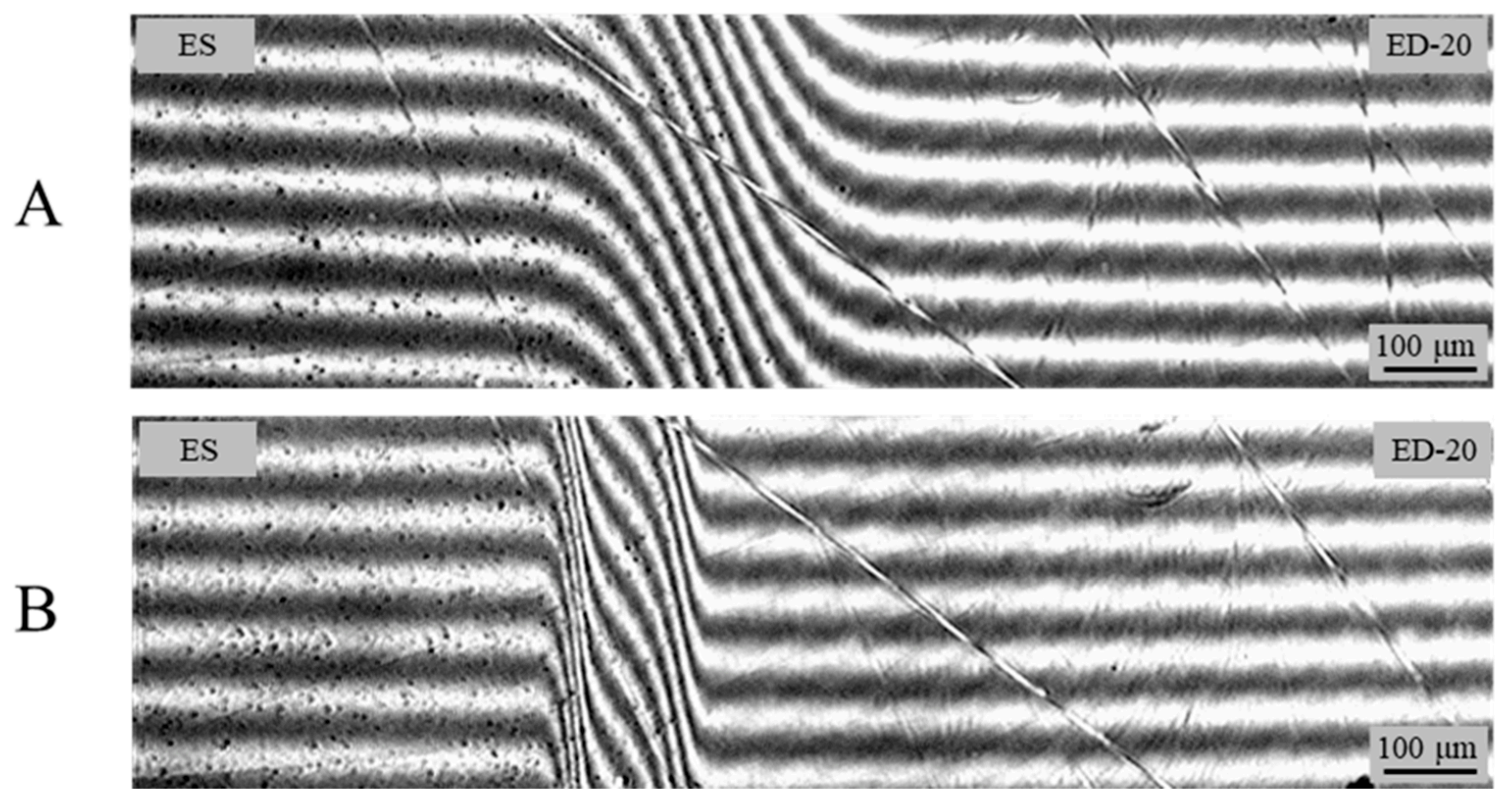
Interferograms of the diffusion zone of the ES−ED-20 binary system at 160 °C (**A**) and at 22 °C (**B**).

**Figure 4:**
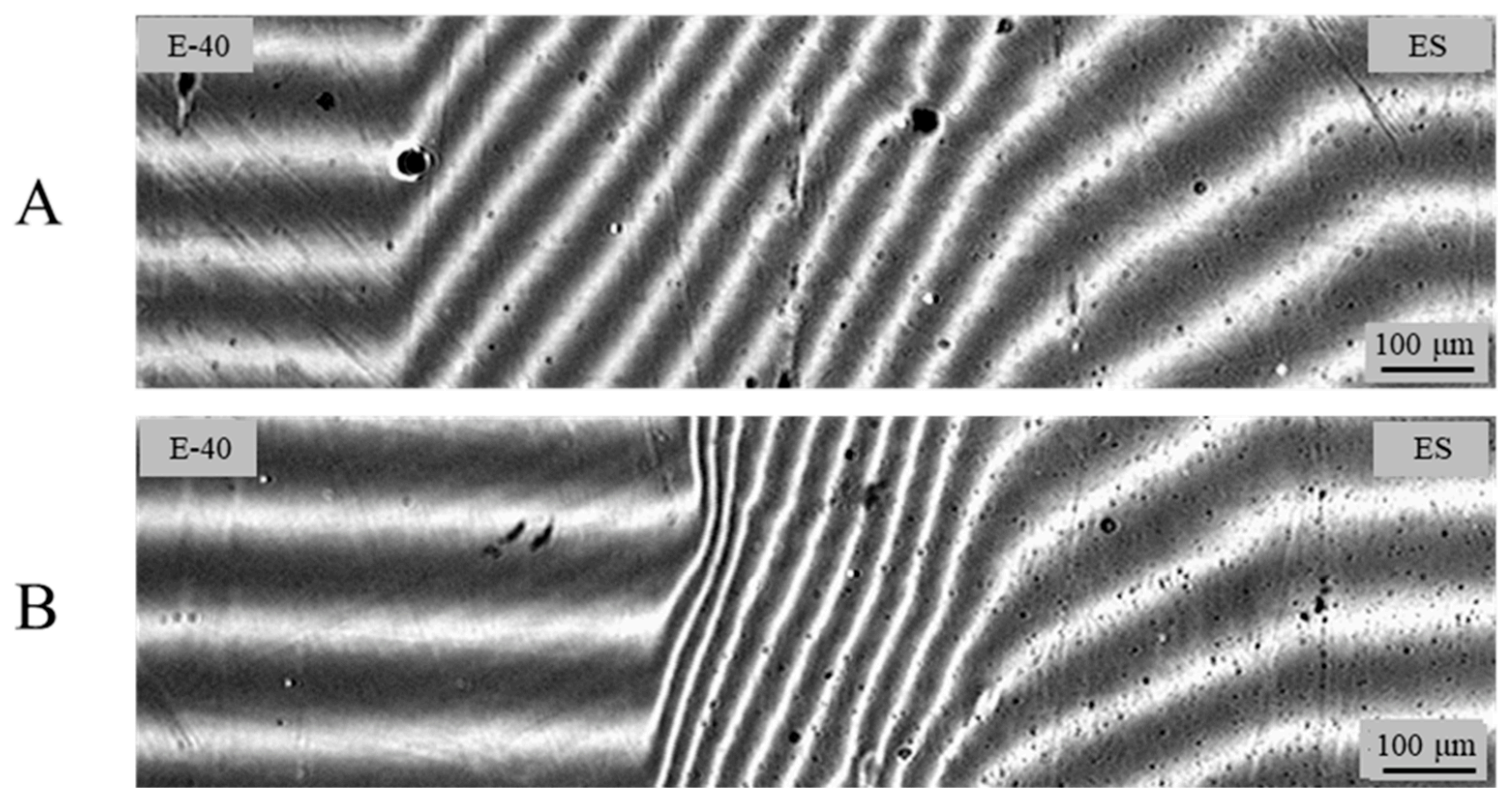
Interferograms of the diffusion zone of the ES−E-40 binary system at 150 °C (**A**) and at 22 °C (**B**).

**Figure 5:**
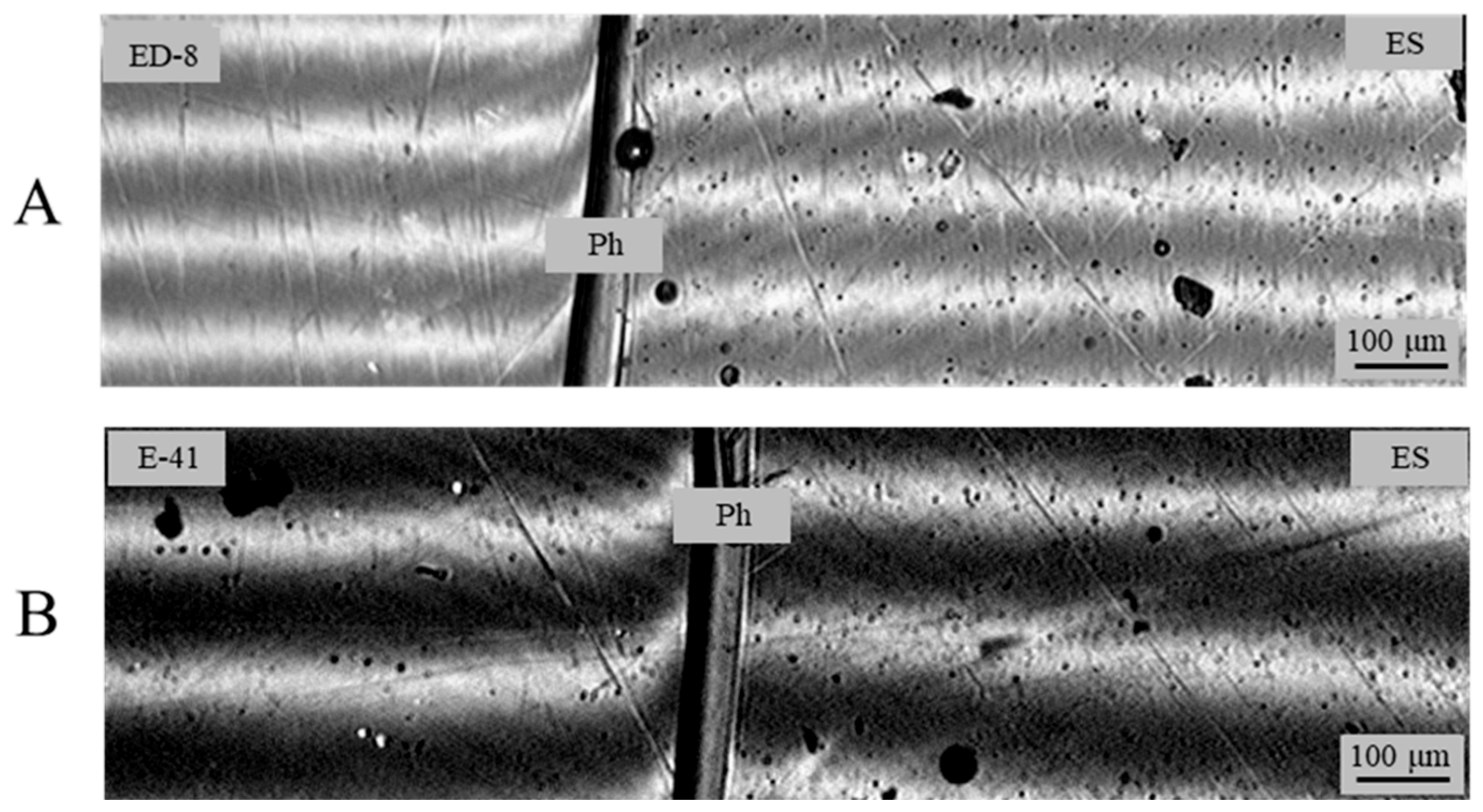
Interferograms of the diffusion zone of the ES−ED-8 (**A**) and ES-E-41 (**B**) binary systems at 90 °C and at 150 °C, respectively. Here, “Ph” is the phase boundary.

**Scheme 2:**
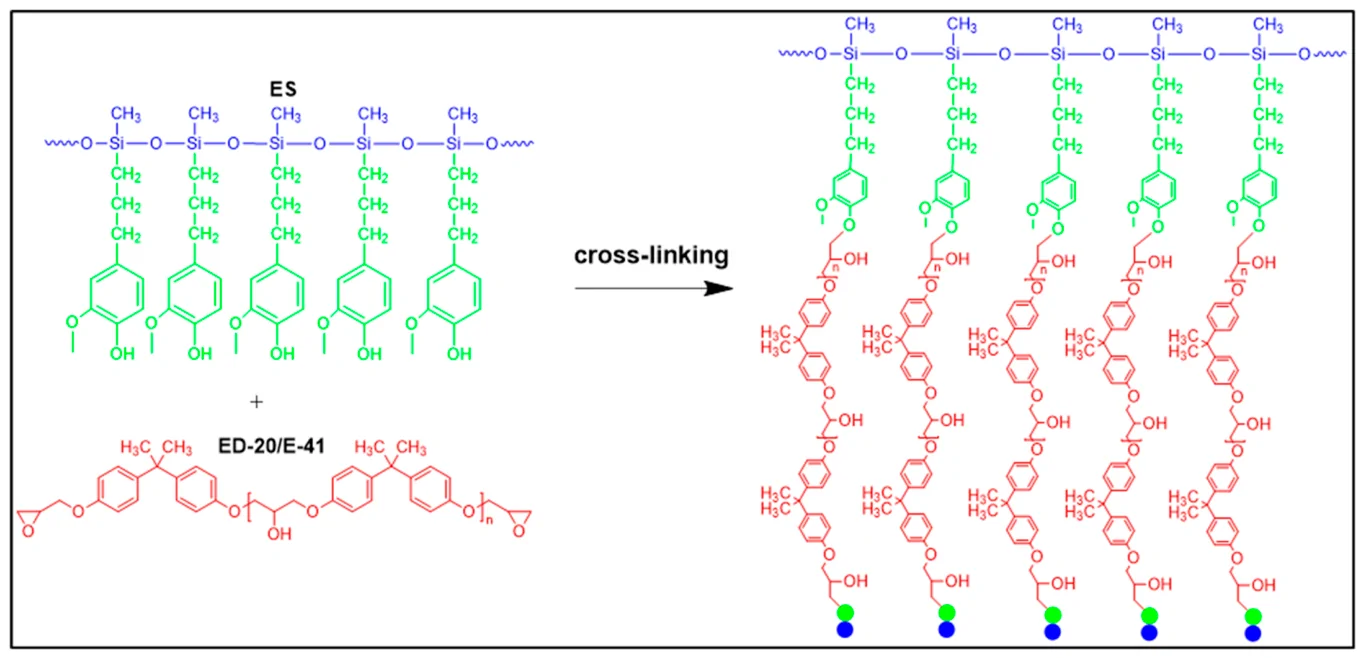
Cross-linking reaction between ES and epoxy resins.

**Figure 6:**
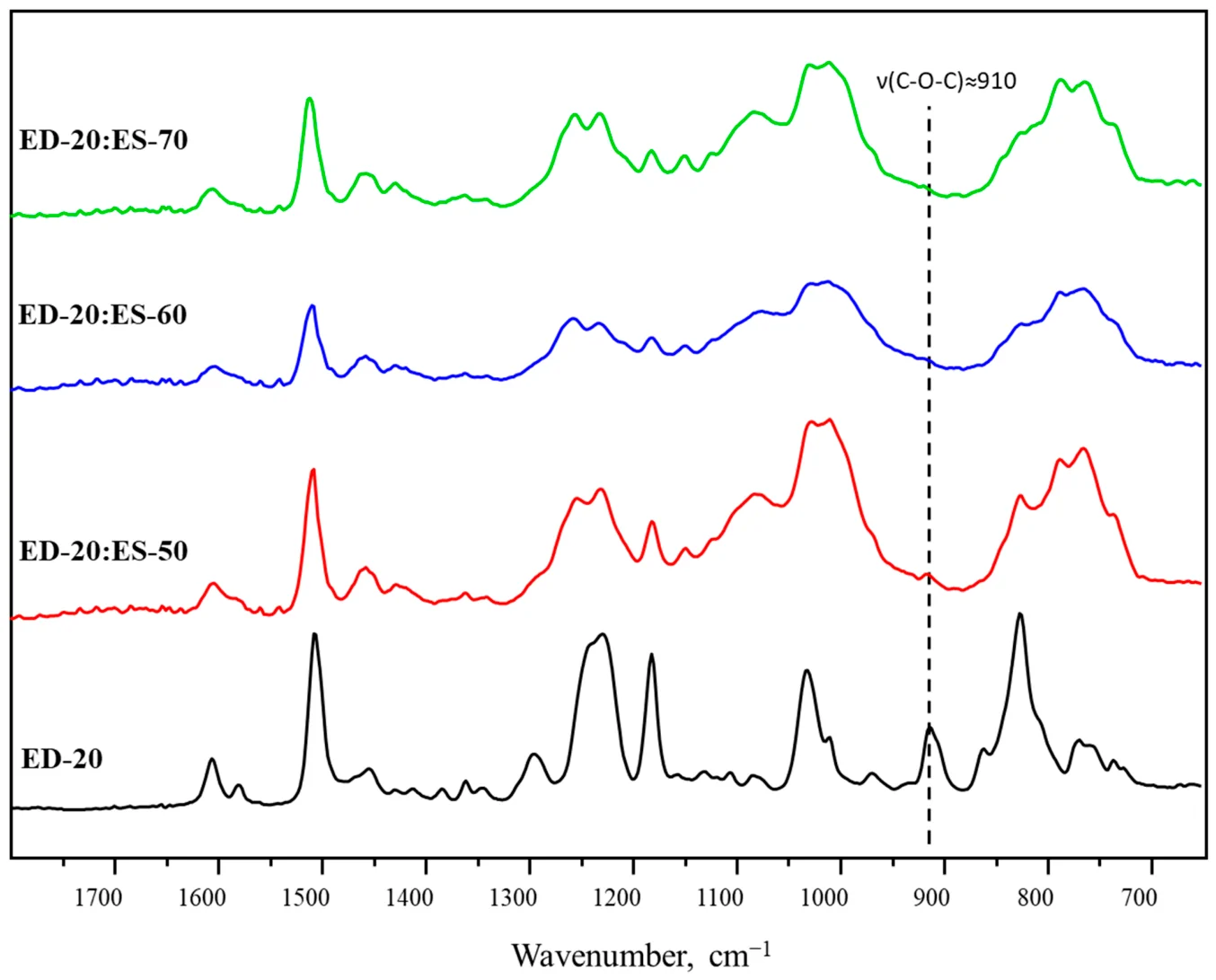
FTIR spectra of the compositions ED-20:ES cured at 180°C.

**Figure 7:**
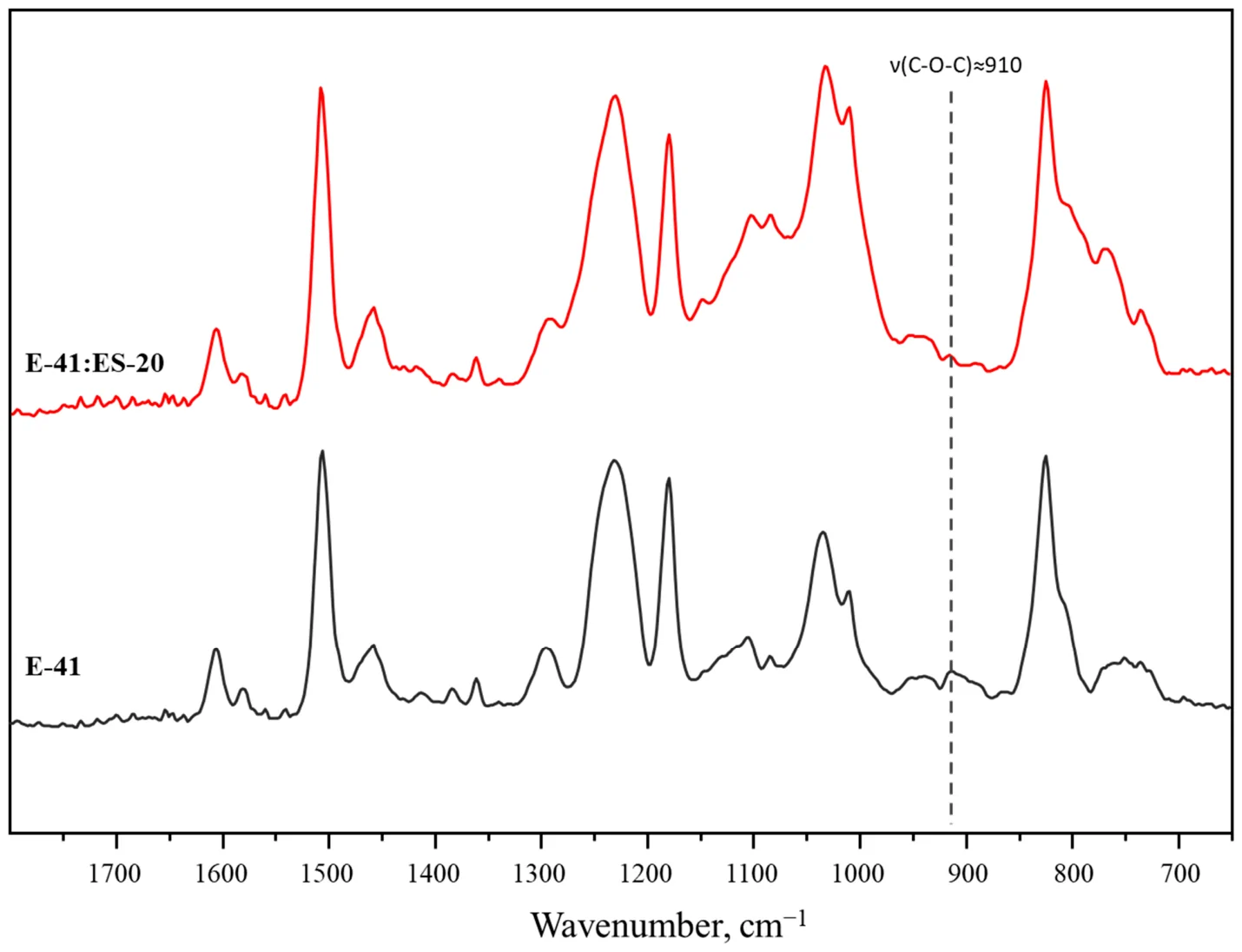
FTIR spectra of the compositions E-41:ES cured at 180°C.

**Figure 8:**
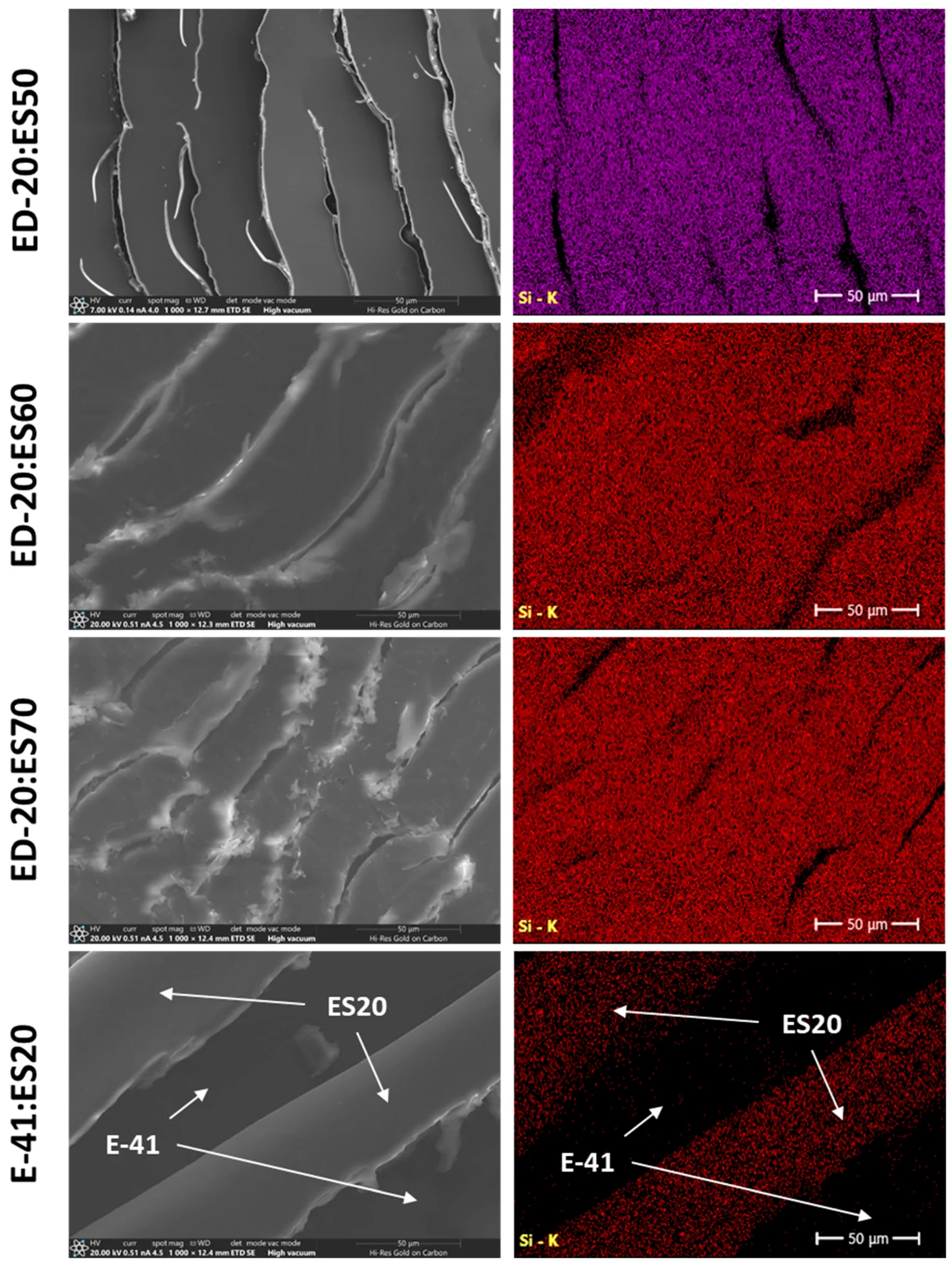
SEM images and Si elemental mapping of the films of cured compositions.

**Figure 9:**
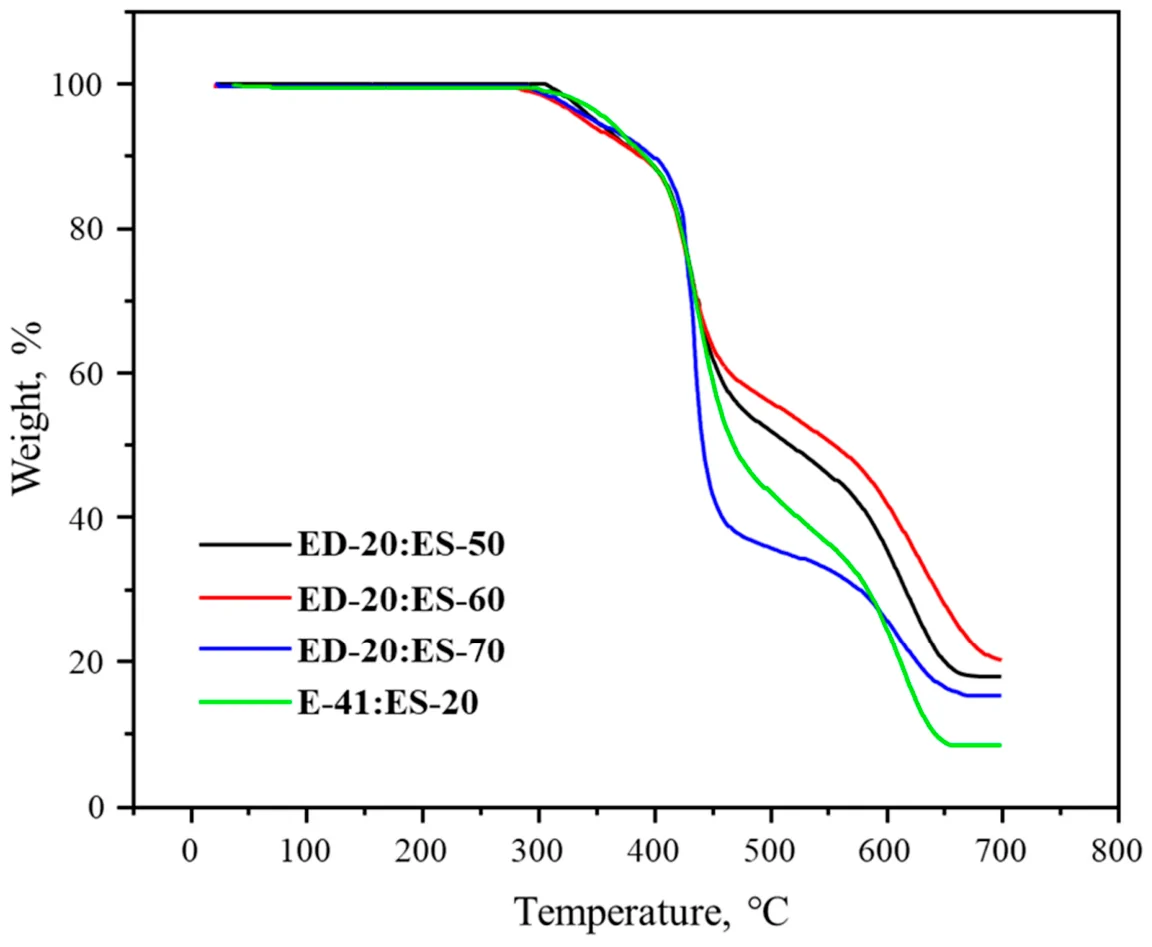
TGA curves of the cured compositions.

**Table 1:** Properties of the used epoxy resins.

Epoxy Resin	Mn	Content of Epoxy Groups, wt. %
ED-20	390–430	20.0–22.5
E-40	570–660	13.0–15.0
ED-8	860–1100	8.0–10.0
E-41	950–1260	6.8–9.1

**Table 2:** Content of the components in the compositions.

Composition	Component Content, wt. %		
	ES	ED-20	E-41
ED-20:ES50	50	50	-
ED-20:ES60	60	40	-
ED-20:ES70	70	30	-
E-41:ES20	20	-	80

**Table 3:** TGA results of the cured compositions.

Composition	T_d5%_, °C	Char Yield at 700 °C, wt. %
ED-20:ES-50	349	18
ED-20:ES-60	339	20
ED-20:ES-70	347	15
E-41:ES-20	360	8

**Table 4:** Physico-chemical and physico-mechanical properties of the obtained coatings.

Parameter	Value			
	ED-20:ES-50	ED-20:ES-60	ED-20:ES-70	E-41:ES-20
Coating thickness, μm	21	18	23	20
Water contact angle, °	83 ± 2	92 ± 2	89 ± 1	90 ± 2
Adhesion by cross-cut test, class	0	0	0	0
Adhesion by pull-off test, MPa	3, 60% A/B, 40% Y/Z	3, 40% A/B, 60% Y/Z	3, 50% A/B, 50% Y/Z	2, 70% A/B, 30% Y/Z
Impact strength, cm	50	50	50	50
Bending strength, mm	1	1	1	1
Pendulum hardness (König), rel. units	0.64	0.87	0.53	0.92
Stability to salt spray at 35 ± 2 °C (10 days), corrosion spreading from the incision, mm	0.2	0.4	0.3	0.1
Coating stability to 3% NaCl solution at 20 ± 2 °C, h	96	96	96	96
Coating stability to distilled water at 20 ± 2 °C, h	120	120	120	120

## Data Availability

The original contributions presented in this study are included in the article and Supplementary Materials. Further inquiries can be directed to the corresponding author.

## Supplementary Materials

The following supporting information can be downloaded at https://www.mdpi.com/article/10.3390/polym18192313/s1. Figure S1. ^13^C-NMR spectra of initial PMHS and eugenol and the final ES. Figure S2. GPC curve of the ES. Figure S3. ^1^H-NMR spectra of ED-20:ES systems after mixing of the components at 125 °C. Figure S4. GPC curves of ED-20:ES systems after mixing of the components at 125 °C. Figure S5. Coatings, based on the ED-20:ES-60 composition, cured at 180 °C: (a) view of the coating made with the use of a pneumatic spray gun; (b) adhesion by a cross-cut test; (c) bending elasticity test; (d) impact strength test. Figure S6. Water droplets on the surfaces of the cured coatings. Figure S7. Corrosion spreading on steel plates after a salt-spray test and removal of the coatings.
