# Supplementary material for: Preparation of Eugenol-Containing Epoxy–Siloxane Polymers for Coating Materials
Source: Polymers (Basel). 2026 Sep 22;18(19):2313. doi: 10.3390/polym18192313 (PMC13667080; doi:10.3390/polym18192313)
Supplement: Supplementary file 1 [file polymers-18-02313-s001.zip › polymers-4553576-supplementary.pdf]

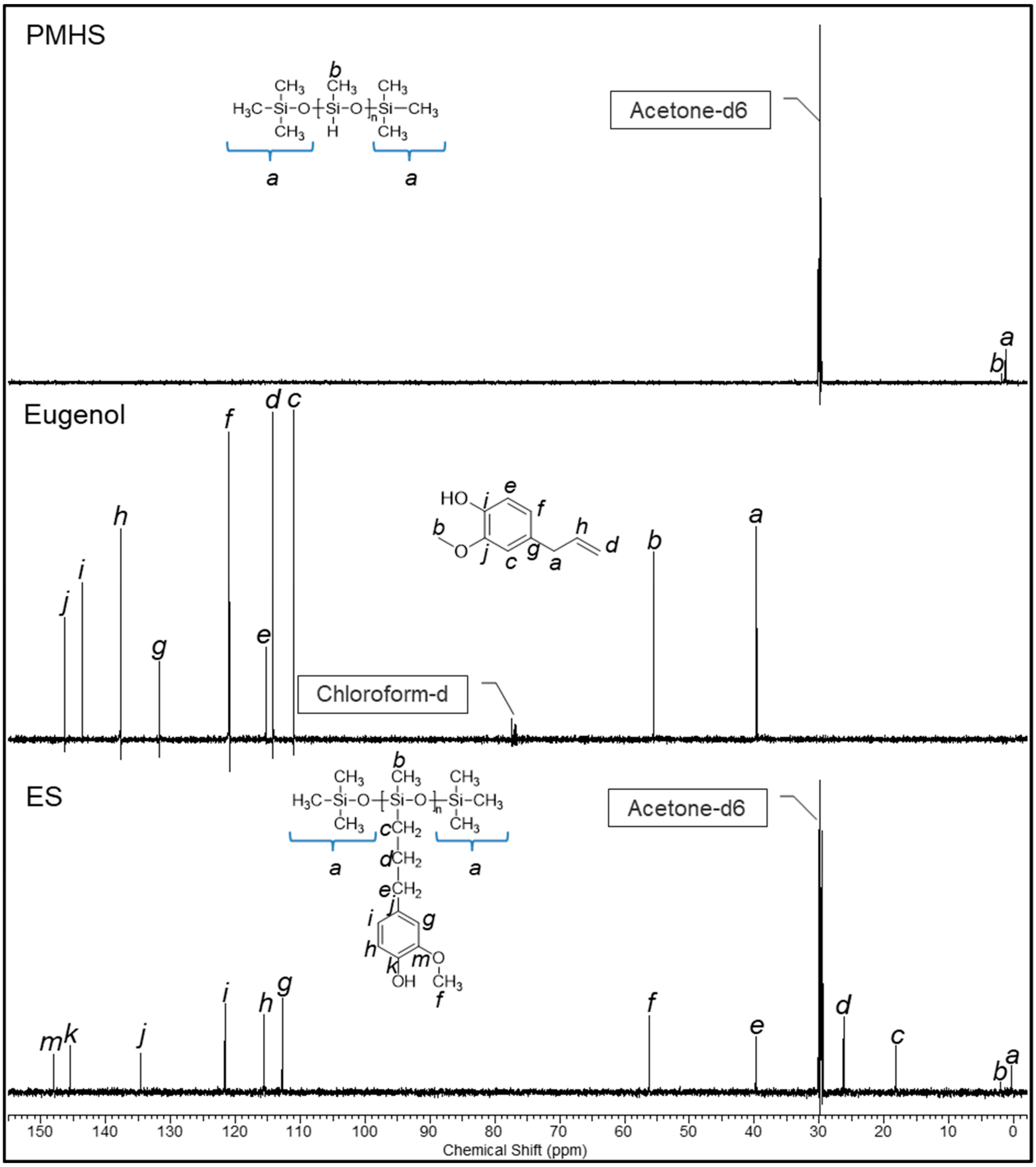

Figure S1. ^{13}C-NMR spectra of initial PMHS and eugenol and the final ES

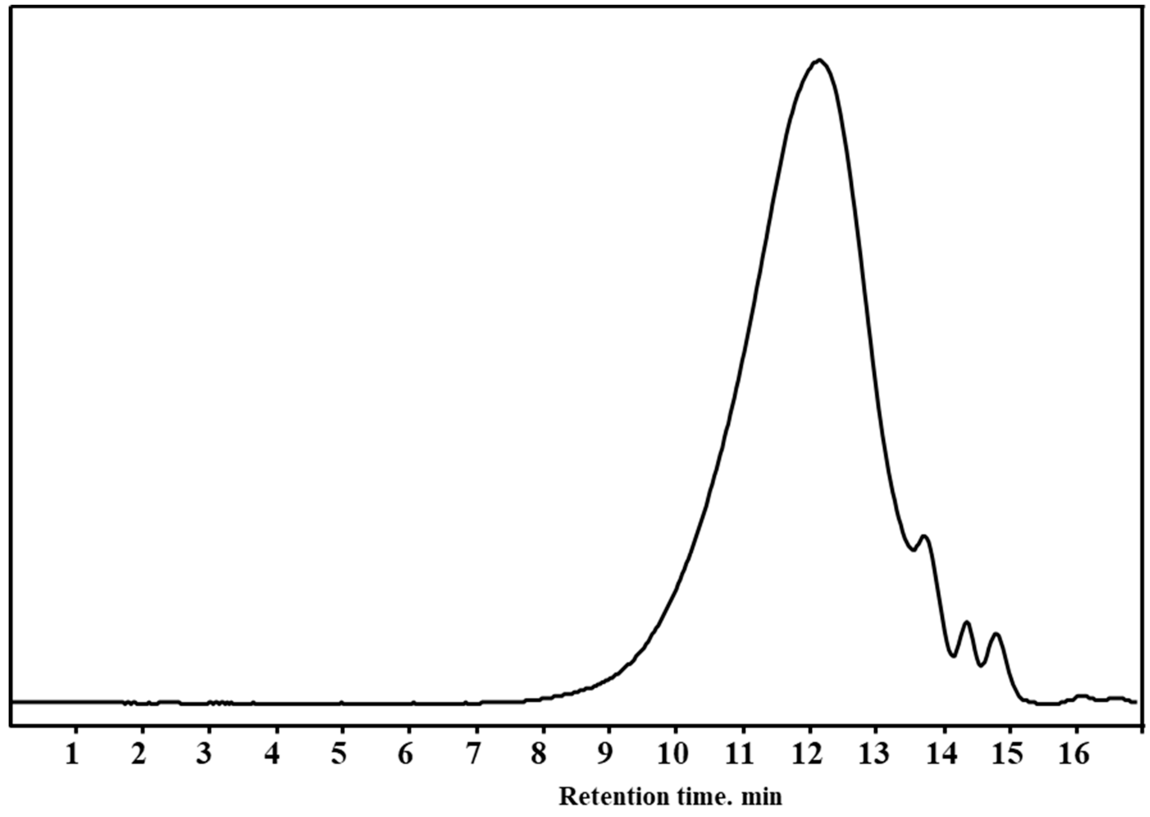

Figure S2. GPC curve of ES.

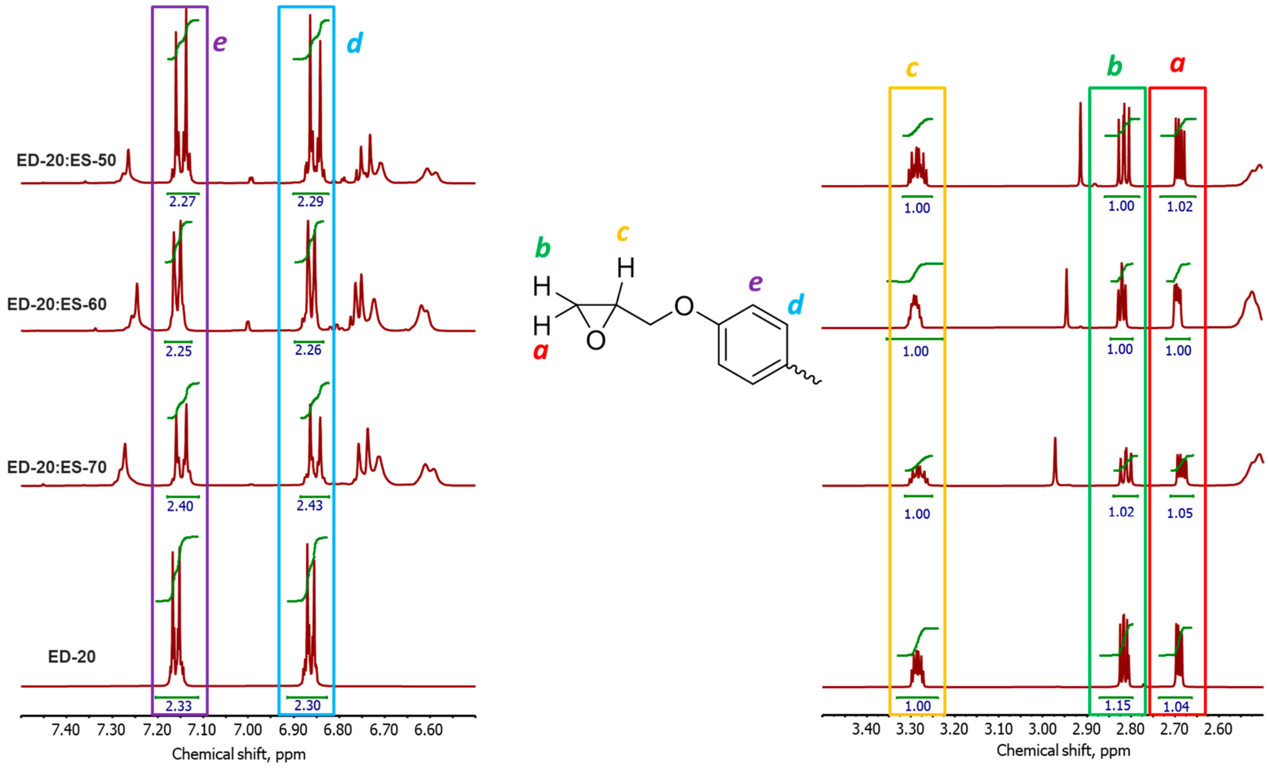

Figure S3. ^{1}H-NMR spectra of ED-20:ES systems after mixing of the components at 125 °C

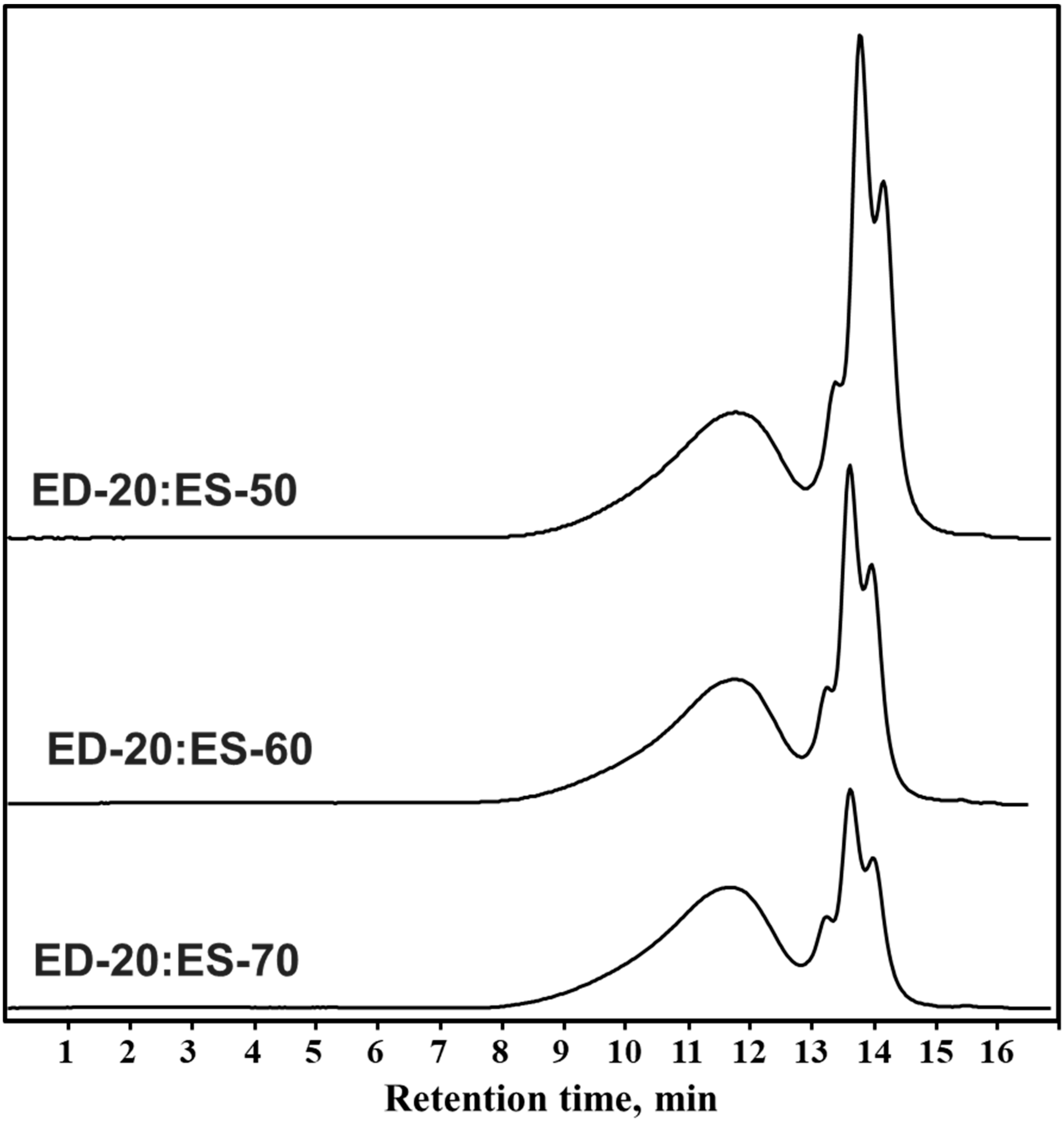

Figure S4. GPC curves of ED-20:ES systems after mixing of the components at 125 °C

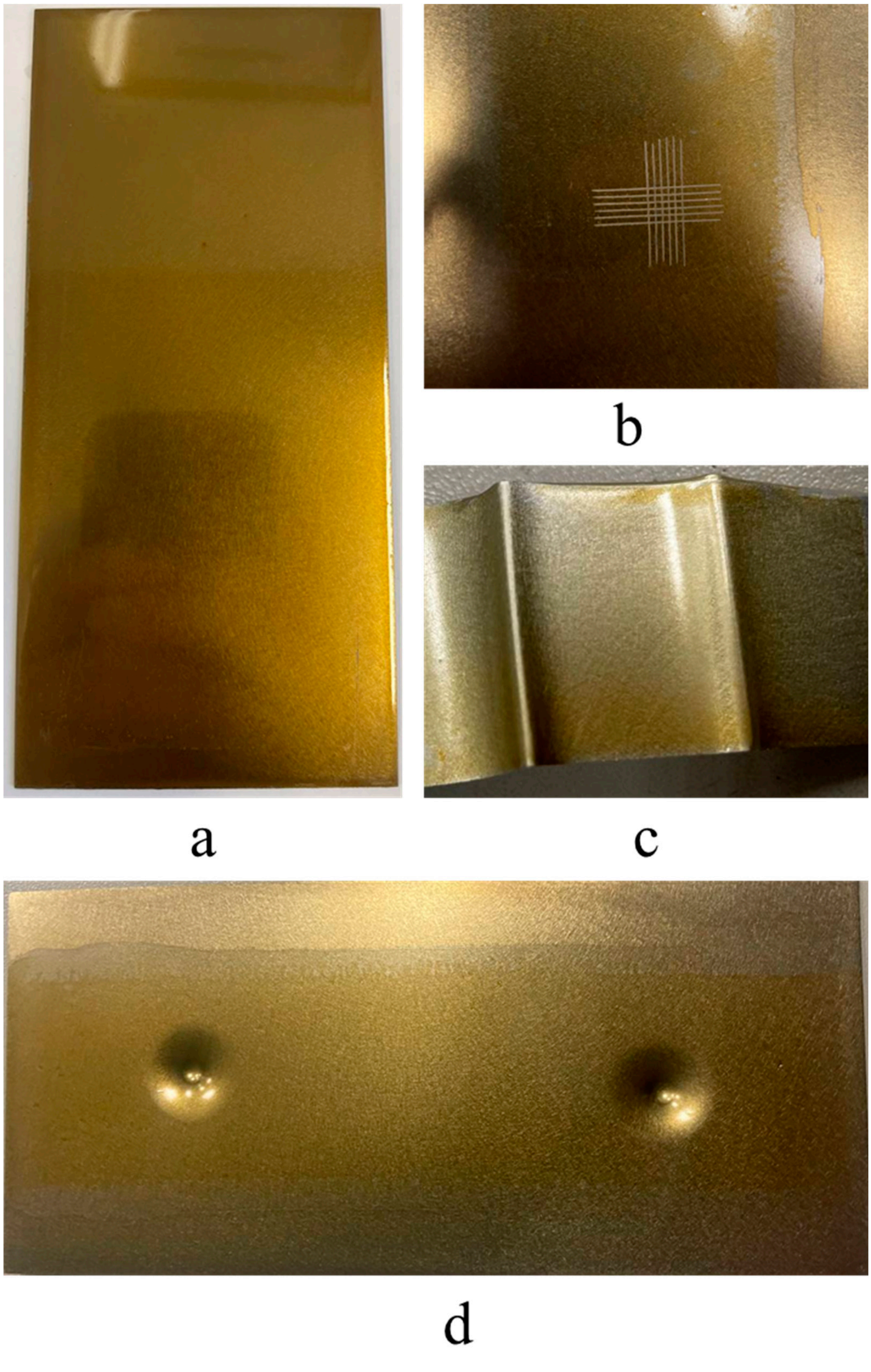

Figure S5. Coatings, based on ED-20:ES-60 composition, cured at 180 °C: a) view of the coating made with the use of pneumatic spray gun; b) adhesion by cross-cut test; c) bending elasticity test; d) impact strength test.

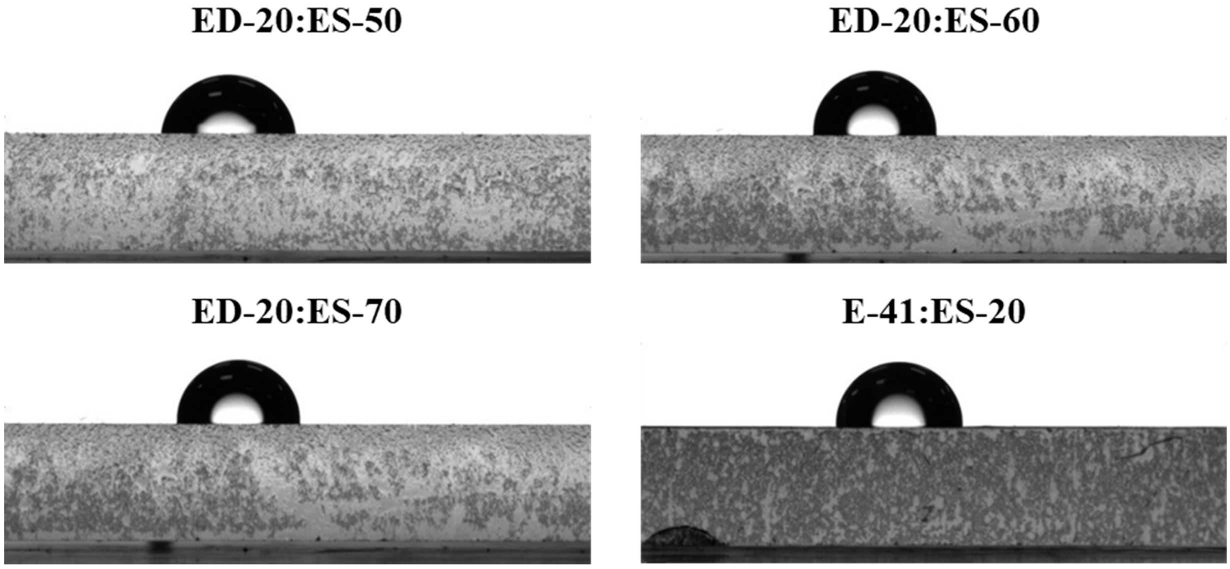

Figure S6. Water droplets on the surfaces of the cured coatings.

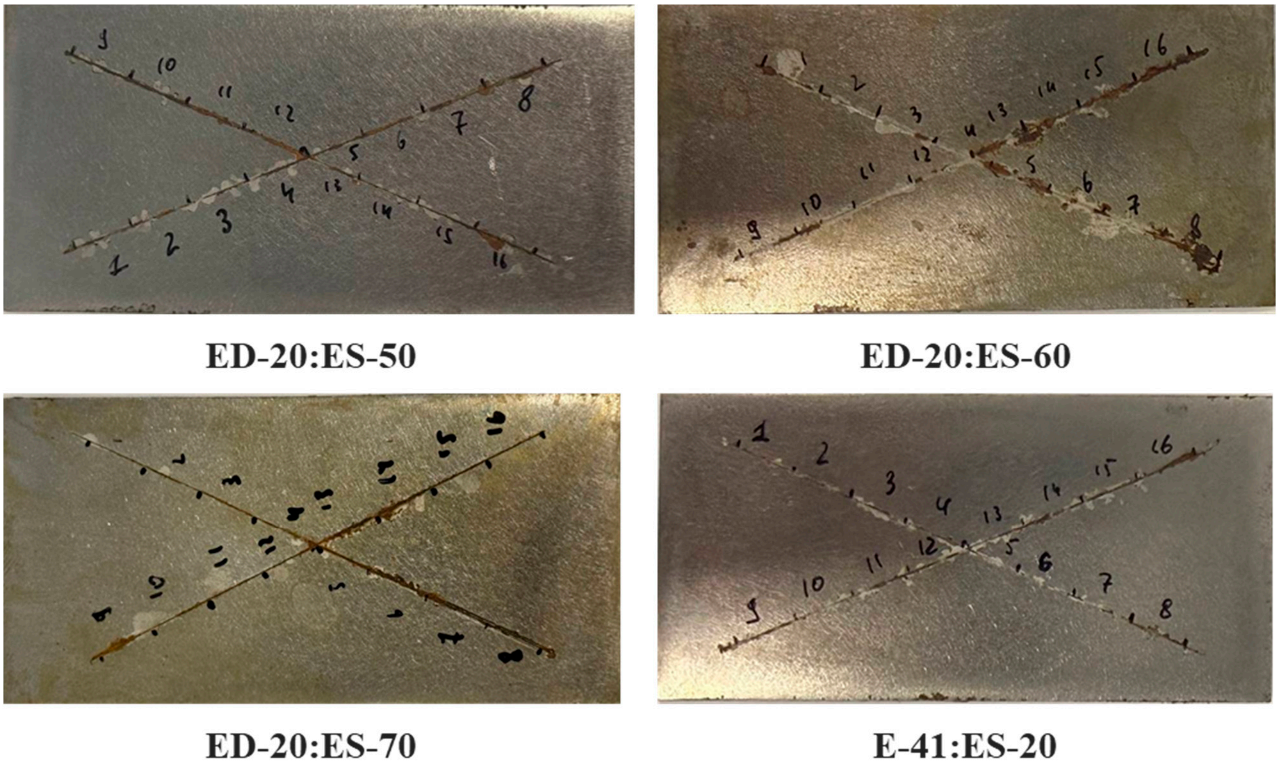

Figure S7. Corrosion spreading on steel plates after salt spray test and removal of the coatings
